# Atypical RXLR effectors are involved in *Phytophthora cactorum* pathogenesis

**DOI:** 10.1007/s42994-025-00198-4

**Published:** 2025-01-27

**Authors:** Zeyang Zheng, Juanjuan Liu, Wenzhong You, Jun Sun, Kehan Wang, Xuemei Zhang, Xinyu Yan, Zhenpan Liu

**Affiliations:** 1 Liaoning Institute of Economic Forestry, Dalian, 116031 China; 2 Liaoning Academy of Agricultural Sciences, Shenyang, 110161 China

**Keywords:** *Phytophthora*, RXLR effector, Gene evolution, Pathogenicity

## Abstract

RXLR effectors are pathogenic factors secreted from oomycetes to manipulate the immunity of the host. Typical RXLR effectors contain an RXLR-dEER motif at the N-terminus, whereas atypical RXLRs show variations on this motif. The oomycete *Phytophthora cactorum* is known to infect over 200 plant species, resulting in significant agricultural economic losses. Although genome-wide identification and functional analyses of typical RXLRs from *P. cactorum* have been performed, little is known of atypical PcaRXLRs. Here, we identified RXLRs, both typical and atypical, in *P. cactorum* and compared them with those of other oomycete pathogens. Fewer RXLRs were identified in *P. cactorum* compared with other *Phytophthora* species, possibly due to fewer duplication events of RXLRs. In contrast, the percentage of atypical RXLRs was higher in *P. cactorum* than in other species, suggesting significant roles in *P. cactorum* pathogenesis. Analysis of RXLR gene expression showed that most were transcribed, suggesting their functionality. Transient expression of two atypical RXLRs in *Nicotiana benthamiana* showed that they induced necrosis dependent on host SGT1 and HSP90. Furthermore, two additional atypical RXLRs suppressed the defense response in *N. benthamiana* and promoted *P. cactorum* infection. These results demonstrate the vital role of atypical RXLRs in *P. cactorum* and provide valuable information on their evolutionary patterns and interactions with host plants.

**Supplementary Information:**

The online version contains supplementary material available at 10.1007/s42994-025-00198-4.

## Introduction

Plants and their microbial pathogens have co-evolved (Ngou et al. 2022). Upon pathogen infection, defence responses are triggered by the plant through its recognition of conserved pathogen-associated molecular patterns (PAMPs) and avirulence effectors (Chang et al. 2022). Pathogens, in turn, release a variety of effector proteins to inhibit the immune response of the host and, thus, facilitate infection (Wang et al. 2022a; Wilson and McDowell 2022; Fernandez 2023). One such effector group is that of the RXLR proteins, which were originally described in oomycetes. These proteins contain a specific motif, the RXLR-dEER motif, or variations thereof, at their N-termini which is responsible for facilitating entry to the host cell, together with C-terminal regions that vary according to the specific protein and are responsible for effector functions (Dou and Zhou 2012; Dong and Ma 2021; Li et al. 2021). These effector functions may lead to plant cell death. For instance, an analysis of RXLR effector proteins from *Phytophthora sojae* found that 11 of these were able to induce cell death in *Nicotiana benthamiana*, together with leaf chlorosis and mottling (Wang et al. 2011). These RXLR may thus be avirulence proteins that exhibit gene-for-gene interactions with specific host resistance proteins, leading to hypersensitive responses in plants (Dalio et al. 2021; Huang et al. 2023).

Certain RXLR proteins contain degenerate dEER motifs and are pathogenic effectors, called atypical RXLRs. For example, an RXLR protein from *Saprolegnia parasitica,* an oomycete that infects fish, lacks the N-terminus dEER motif and acts by entry into host cells to promote virulence (Wawra et al. 2012). The majority of RXLRs from *Pythium* fall into the atypical category and contain degenerate dEER motifs (Ai et al. 2020). These proteins are able to induce either necrosis or defense responses in plants (Ai et al. 2020). *Phytophthora* species express numerous functional RXLRs that have degenerate dEER motifs. One well-documented example is Avr1b from *P. sojae* which although it lacks the typical dEER motif, requires its N-terminal region to deliver proteins into plant cells (Dou et al. 2008). The *P. infeastans* proteins, Avr1 and PexRD2, are atypical RXLRs that are closely associated with plant immunity (King et al. 2014; Du et al. 2015). Thus, atypical RXLRs are one of the vital components of *Phytophthora* pathogenesis.

The phytopathogenic oomycete *Phytophthora cactorum* is able to infect numerous plants from a variety of families. It is known to cause rot of the plant roots, collar, and crown, together with infecting both leaves and fruit, and is recognized as the 22st most important oomycete pathogen (Kamoun et al. 2015; Chen et al. 2023) affecting numerous crops, such as apple, pear, and strawberry, and ornamental plants such as the rhododendron (Chen et al. 2014, 2018). Furthermore, *P. cactorum* is homothallic and its sexual oospores are capable of long-term survival in soils, representing a significant challenge for control (Chen et al. 2014, 2018). Although genome-wide identification and functional analyses of typical RXLRs from *P. cactorum* have been undertaken (Chen et al. 2014; Gogoi et al. 2023), little is known of its atypical PcaRXLRs.

Plants use sophisticated systems to resist attack by exogenous pathogens. These involve two main strategies, namely, PAMP-triggered immunity (PTI) and effector-triggered immunity (ETI) (Jones and Dangl 2006; Locci and Parker 2024). In the former, cell surface receptors recognize pathogen-derived PAMPs for basal immunity and trigger downstream responses, such as the production of ROS (Jones et al. 2024). However, many pathogens have evolved escape mechanisms, including the secretion of effectors against these receptors, allowing them to modulate the immunity of the host (Ruiz-Bedoya et al. 2023). As a countermeasure, plants have evolved ETI-associated strategies using nucleotide-binding leucine-rich repeat (NLR) proteins that can detect these microbial effectors, either directly or indirectly (Shi et al. 2020; Contreras et al. 2023). Relative to PTI, the response of ETI is stronger and can involve the death of the cell, a characteristic feature of the hypersensitive response (HR) (Yu et al. 2024). Some NLR functions are mediated by the conserved chaperone complex of SGT1 and HSP90 (Shirasu 2009; Kadota et al. 2010). Examples include Rx, an agronomically important sensor NLR from potato that confers resistance to Potato virus X by recognizing its coat protein (CP)(Botër et al. 2007), and Mla6, which confers barley resistance to the powdery mildew pathogen *Blumeria graminis f. sp. hordei* (Azevedo et al. 2002).

Here, we analyzed RXLRs in *P. cactorum*, including both typical and atypical types, and compared them with those of other oomycete pathogens. Fewer RXLRs were identified in *P. cactorum* compared with other *Phytophthora* species, probably due to fewer duplication events in the RXLRs of *P. cactorum*. In contrast, the percentage of atypical RXLRs in *P. cactorum* was higher than that in other species, suggesting their important roles in *P. cactorum* pathogenesis. Analysis of the expression profiles of atypical RXLR genes showed that most were transcribed, suggesting they are true functional genes. Transient screening assays in *Nicotiana benthamiana* found that two atypical RXLRs induced necrosis in conjunction with host SGT1 and HSP90. Furthermore, two additional atypical RXLRs were observed to suppress the defence response in *N. benthamiana* and promote *P. cactorum* infection. Overall, the findings showed that atypical RXLRs have critical functions in promoting infection by *P. cactorum*, and provide valuable information on their evolution and interactions with host plants.

## Results

### Identification of RXLRs in *P. cactorum*

**Fig. 1:**
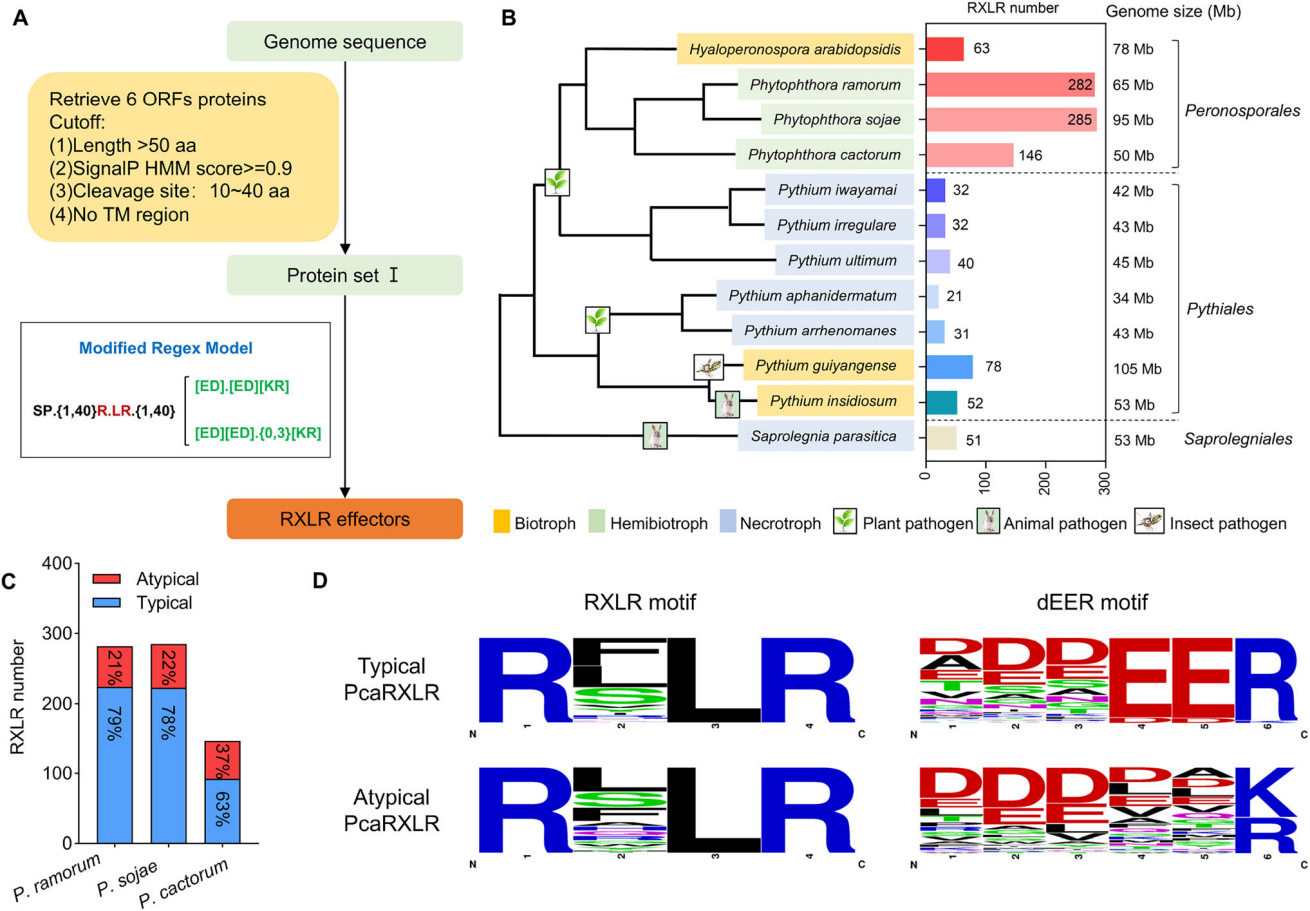
Identification of PcaRXLRs. **A** Process of identifying RXLR effectors. Protein set I included ORFs that encoded secreted proteins (containing a signal peptide [SP]) and lacking a transmembrane domain (TM). **B** Prediction of RXLRs. Phylogenetic relationships were obtained from the taxonomy database and results of earlier investigations. Boxplots indicate the number of RXLR genes per species, with genome sizes indicated after the boxplot. **C** The numbers and ratios of atypical/typical RXLRs in three *Phytophthora* species. **D** Weblogo of the RXLR and dEER motif of typical and atypical PcaRXLRs

To identify RXLR effectors in *P. cactorum*, we used a previously reported RXLR prediction pipline to screen the entire sequenced genome of *P. cactorum* (Fig. 1A) (Ai et al. 2020). This prediction pipline employed a loose criterion for dEER motif identification, allowing the prediction of both RXLRs containing precise dEER motif (EER pattern, hereafter typical RXLRs), and RXLRs containing degenerate dEER motifs ([ED].[ED][KR] or [ED][ED].{0,3}[KR] pattern; hereafter atypical RXLRs). Using this pipline for RXLR prediction is rational, as many functionally verified RXLRs contain degenerate dEER motifs, including Avr1 and PexRD2 family RXLRs from *P. infestans* and PsAvr1b from *P. sojae* (Ai et al. 2020). The well-studied genomes of *P. cactorum*, *P. sojae, P. ramorum*, *H. arabidopsidis*, *Saprolegnia parasitica*, and seven *Pythium* species were used as references (Tables S1, S2).

In this RXLR prediction pipline, all open reading frames (ORFs) were initially identified in the genomes as all documented *RXLRs* lack introns (Jiang et al. 2008). The sequences were then classified as protein set I if encoding proteins included signal peptides (SPs) but not transmembrane domains (TMs) (Fig. 1A). The protein set I sequences that match the modified regex model (SP.{1,40}R.LR.{1,40}[ED].[ED][KR] or SP.{1,40}R.LR.{1,40}[ED][ED].{0,3}[KR]) were classified as RXLR effectors. Our prediction identified 63 RXLRs in *H. arabidopsidis*, 282 in *P. ramorum*, and 285 in *P. sojae* (Fig. 1B). These numbers align with previous reports (Ai et al. 2020), validating the robustness of our prediction pipeline. Notably, we detected only 146 RXLR effectors in *P. cactorum*, fewer than in other *Phytophthora* species. Nevertheless, this number still exceeds the RXLR effector counts found in species belonging to *Hyaloperonospora*, *Pythiales*, and *Saprolegniales* (Fig. 1B).

Interestingly, although fewer RXLRs were identified in the *P. cactorum* genome, these included a greater percentage of atypical RXLRs in *P. cactorum* RXLRs compared with in *P. ramorum* and *P. sojae* (Fig. 1C). *P. ramorum* and *P. sojae* had 21% and 22% atypical RXLRs in their RXLR reservoir, respectively. In contrast, 37% of the RXLRs in *P. cactorum* were atypical (Fig. 1C), possibly indicating that atypical RXLRs are more important for *P. cactorum* pathogenesis relative to the other species. We found that the RXLR motifs were similar between the two types of effectors. The variable amino acid positions in RXLR motifs were predominantly occupied by phenylalanine (F), serine (S), or leucine (L) residues in both of the two type RXLRs (Fig. 1D). While both types of RXLR effectors were enriched in aspartate (D) or glutamate (E) residues in their dEER motifs, atypical RXLRs lacked the precise EER pattern. Furthermore, atypical RXLRs showed variation in the final residue, with lysine (K) sometimes substituting for arginine (R).

### Genetic relatedness of *P. cactorum* RXLRs with other oomycete pathogens

**Fig. 2:**
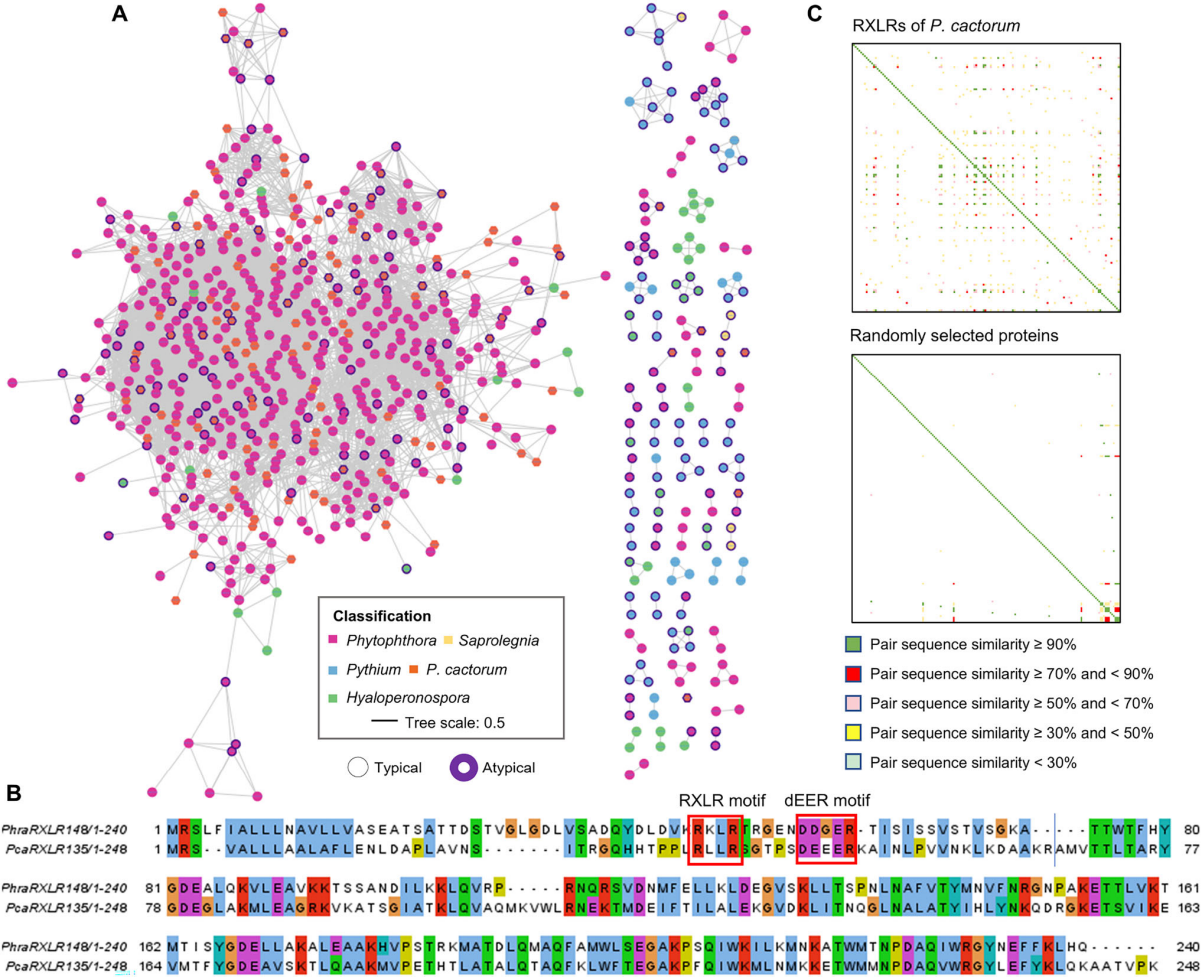
The relatedness between PcaRXLRs and other oomycete RXLRs. **A** Network showing the relatedness of predicted RXLR effectors. Dots indicate individual RXLRs with similar RXLRs (Blastp, e-value < 1e-5) linked by edges. Pink dots represent *Phytophthora* RXLRs from *P. sojae* and *P. ramorum*. Atypical RXLRs are labelled by purple boarder. **B** Sequence alignment of PhraRXLR148 and PcaRXLR135. RXLR motif and dEER motif are labelled by red boxes. **C** Relationship between *P. cactorum* RXLRs. Pairwise similarity of *P. cactorum* RXLRs is shown at the top and that of randomly selected proteins is shown at the bottom

Significant genetic relatedness was observed between RXLR effectors from *P. sojae* and *P. ramorum*, indicating common ancestry (Jiang et al. 2008). The predicted RXLRs from *P. cactorum* were then aligned using BLASTP following removal of the highly similar SPs and an *e*-value < 1e-5 (commonly accepted threshold for ortholog detection). This showed that 940 RXLR proteins had significant hits to at least one other RXLR. Overall, 12,750 RXLR pairs were found, forming a relatedness network (Fig. 2A). *Phytophthora* RXLRs were found to have greater within-genus relatedness compared with proteins from *Hyaloperonospora* and *Pythium*, suggesting the presence of multiple recent expansions in the *Phytophthora* genome relative to those of *Hyaloperonospora* and *Pythium* (Fig. 2A), and also confirming the reliability of RXLR identification in *P. cactorum*. Interestingly, our analysis revealed close sequence relationships between atypical and typical RXLRs, suggesting potential evolutionary transitions between these two forms. For example, *P. ramorum* PhraRXLR148 shares high sequence similarity with *P. cactorum* PcaRXLR135, despite their different RXLR characteristics: PhraRXLR148 is an atypical RXLR with a degenerate dEER motif (DDGER), while PcaRXLR135 is a typical RXLR containing the canonical DEEER motif (Fig. 2B).

We investigated sequence relationships among *P. cactorum* RXLRs using all-against-all BLASTP analysis with an e-value threshold of 1e-5 combined with sequence similarity coverage. The analysis revealed that PcaRXLRs showed higher sequence relatedness compared to randomly selected proteins. Specifically, 29 PcaRXLRs had homologous pairs with sequence similarity exceeding 70%, while 38 PcaRXLRs showed sequence similarity between 40 and 70% with their closest pairs. The remaining 79 PcaRXLRs displayed sequence similarity below 40% (Fig. 2C). These findings suggest limited duplication events during the evolution of PcaRXLRs.

### *P. cactorum* RXLRs underwent fewer tandem duplication events compared with other *Phytophthora* species

**Fig. 3:**
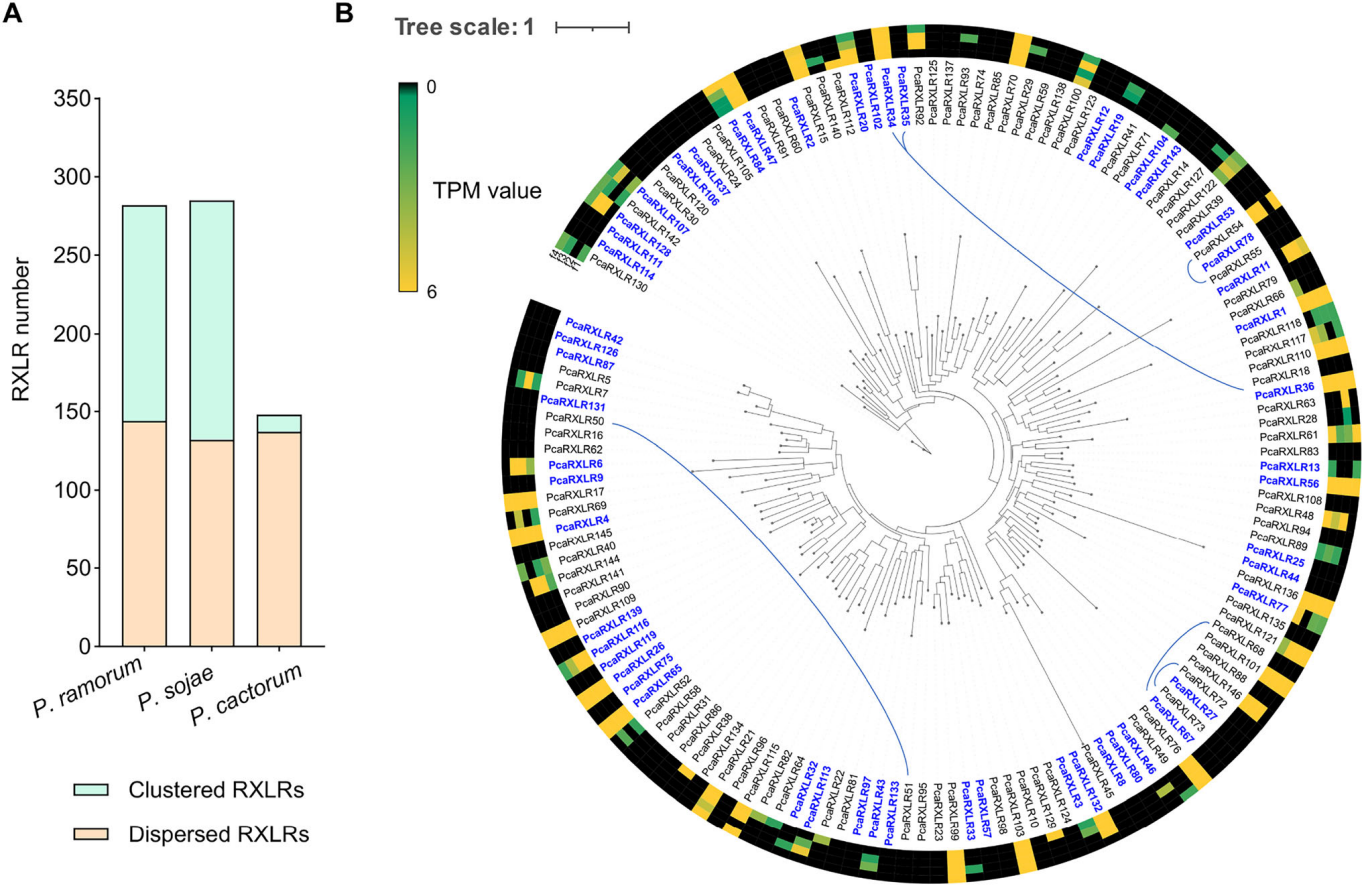
The evolutionary and transcriptome patterns of PcaRXLRs. **A** Less clustered RXLRs encoded by *P. cactorum*. RXLRs located within 20 kb of each other in the genome are considered clustered RXLRs, while the remaining RXLRs distributed elsewhere in the genome are dispersed RXLRs. **B** Transcription level of RXLRs in 4 *P. cactorum* isolates. The phylogenetic tree of PcaRXLRs was constructed using MEGA7 software. Mycelial samples were collected and subjected to RNA extraction for RNA-seq analysis. PcaRXLRs marked in blue indicate atypical RXLRs. The colored outer circles represent TPM (Transcripts Per Million) values calculated from RNA-seq data of four isolates (Phyto01-Phyto04). Clustered PcaRXLRs are linked by blue lines

Larger RXLR reservoir might be resulted from gene tandem duplication and genomic rearrangements. For example, significant gene tandem duplication has been reported in *P. guiyangense* RXLRs (Ai et al. 2020). *Phytophthora* RXLR genes also cluster closely (< 100 kb) in the genome, showing high sequence similarities (Win et al. 2007), indicative of gene duplications. To investigate the reason why the RXLR reservoir of *P. cactorum* is smaller than that of other *Phytophthora* species, we analyzed putative gene duplication events in *P. cactorum* RXLRs. Interestingly, compared with *P. ramorum* and *P. sojae* in which almost half of the RXLRs showed clustering (< 20 kb), minimal RXLR clustering was observed in the *P. cactorum* genome (Fig. 3A). This suggests that the *P. cactorum* genome underwent fewer duplication events during its evolution, which may be the explanation for its smaller RXLR reservoir compared to *P. ramorum* and *P. sojae*. Furthermore, phylogenetic analysis using complete RXLRs from *P. cactorum* showed that the clustered *P. cactorum* RXLRs shared less sequence similarity (Fig. 3B).

### Transcription pattern of *PcaRXLRs*

To investigate the transcription of the predicted *P. cactorum RXLRs*, reported high-quality RNA-seq data from four *P. cactorum* isolates were analyzed to assess the presence of 146 *PcaRXLR* transcripts (Poimala et al. 2021). *PcaRXLRs* with TPM (transcripts per million kilobases) values were consistently < 1 in all isolates were considered non-transcribed genes, with the remaining genes considered transcribed genes. In total, nearly half of the *PcaRXLRs* (70/146) were found to be transcribed genes. In particular, nearly half of transcripts of atypical *PcaRXLRs* (26/54) were detected in one or more of the isolates (Fig. 3B). Interestingly, many *PcaRXLR* transcripts were found in some isolates but were lost in others. For example, transcripts of *PcaRXLR130/142/107* were found in three isolates but were absent in the fourth *P. cactorum* isolate (Fig. 3B), while *PcaRXLR82/64/32* transcripts were observed in one isolate and not in three other isolates. This suggests that the transcription of some *PcaRXLRs* was restricted to specific isolates, consistent with previous observations of high diversity between *RXLR* numbers and transcription between different isolates (Zhang et al. 2019). Collectively, these results indicate that many of the predicted *PcaRXLRs*, especially the atypical *RXLRs*, are truly transcribed.

### PcaRXLR4 and PcaRXLR56 inhibit INF1-mediated cell death and promote *P. cactorum* infection in *N. benthamiana*

**Fig. 4:**
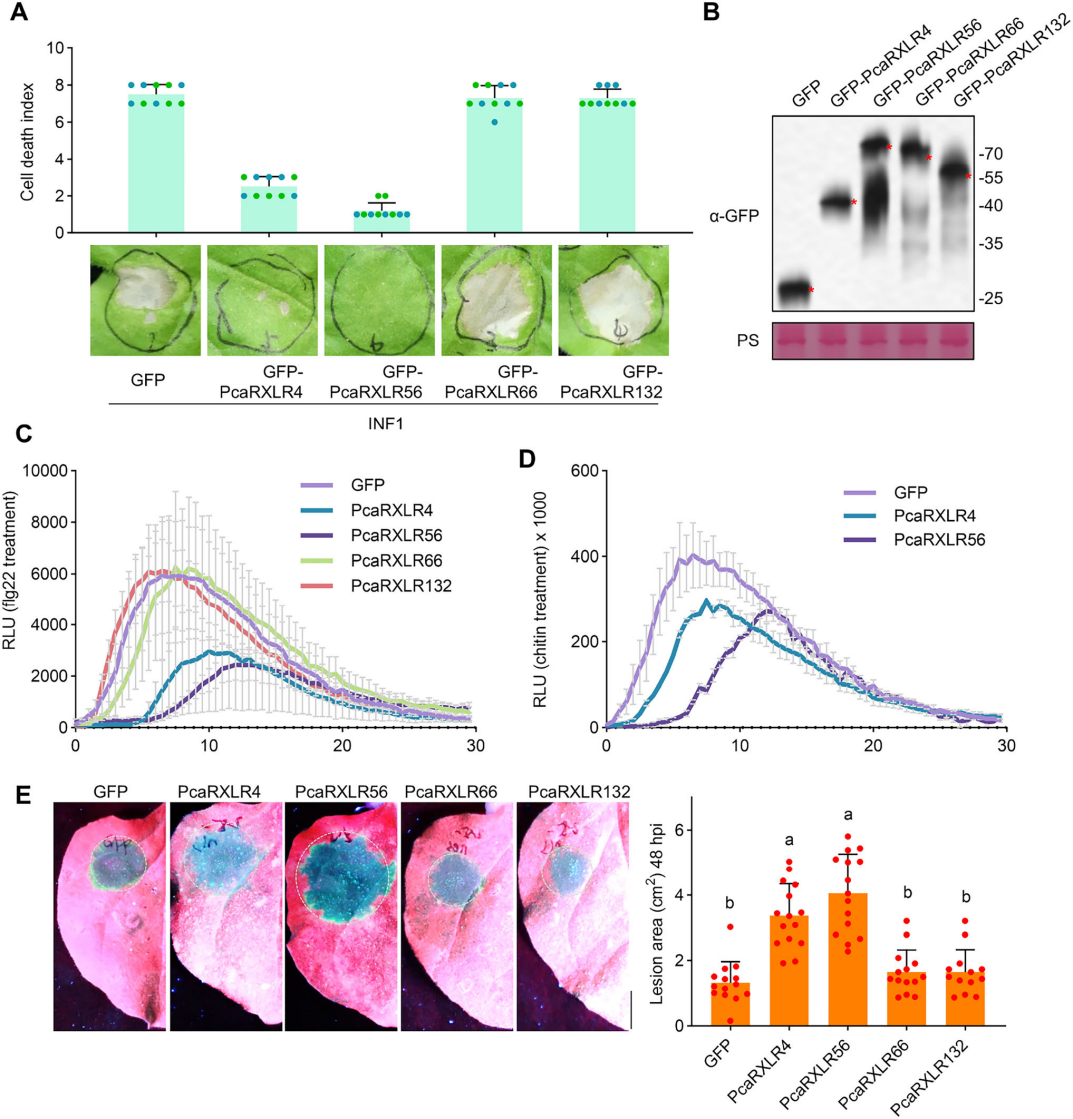
PcaRXLR4 and PcaRXLR56 are involved in virulence of *P. cactorum*. **A** INF1 induced cell death is suppressed by PcaRXLR4 and PcaRXLR56. INF1 was transient expressed with PcaRXLRs in *N. benthamiana*. GFP was used as the negative control. Images on day 6 post-infiltration (dpi). Cell death indexes were calculated and shown above. Two independent biological replicates were labelled using different colors. **B** Immunoblot analysis. The α-GFP (green fluorescent protein) antibody was used to detect expression of the indicated constructs. Equal loading of each sample is indicated by Ponceau S staining (PS) of the RuBisCO protein. **C**–**D** PcaRXLR4 and PcaRXLR56 inhibit ROS bursts induced by Flg22 and chitin. ROS bursts were assessed immediately after leaf treatment with 1 μM flg22 (**B**) or 1 μM chitin (**C**). Data represent mean ± SD from at least 6 replicates. **E** PcaRXLR4 and PcaRXLR56 promotes *P. cactorum* infection in *N. benthamiana* leaves. Indicated proteins were expressed in the leaves of *N. benthamiana,* followed by inoculation with *P. cactorum* after 24 h. Healthy tissue appears red under UV light while infected tissue appears blue. Lesion areas were determined by ImageJ. Data represent mean ± SD (n > 12); significant differences are indicated by lowercase letters, with significance determined by one-way ANOVA at *P* < 0.05). Bar = 1 cm

Next, we asked whether atypical *PcaRXLRs* are involved in *P. cactorum* pathogenesis. INF1 from *P. infestans* is a widely used *Phytophthora* elicitor that can induce plant PTI and subsequent cell death (Derevnina et al. 2016). To determine whether *PcaRXLRs* could suppress host PTI, we co-expressed 17 *PcaRXLRs*, whose transcripts had been detected in at least three isolates (conserved atypical PcaRXLR) and were considered potentially relevant to pathogenesis, with INF1. The cell death index was calculated as previously described (Segretin et al. 2014). No cell death was observed when INF1 was co-expressed with PcaRXLR4 and PcaRXLR56 (Fig. 4A). In contrast, when INF1 was co-expressed with other 15 PcaRXLRs (using PcaRXLR66 and PcaRXLR132 as examples in the figure), cell death was induced in *N. benthamiana* (Fig. 4A). Western blot assay indicated that all proteins expressed correctly (Fig. 4B). These results indicated that PcaRXLR4 and PcaRXLR56 could inhibit INF1-induced cell death but PcaRXLR66 and PcaRXLR132 could not. Furthermore, we investigated whether PcaRXLR4 and PcaRXLR56 could inhibit ROS bursts in the host induced by a well-documented PAMP, flg22 (Sun et al. 2013). In the presence of PcaRXLR4 and PcaRXLR56, flg22-induced ROS bursts were observed to be weaker, with peak values occurring later in comparison with the GFP control and the RXLR PcaRXLR66 and PcaRXLR132 (Fig. 4C). To test whether PcaRXLR4 and PcaRXLR56 could also suppress ROS burst induced by other PAMP, we treated PcaRXLR4 and PcaRXLR56 with chitin. The results demonstrated that both effectors inhibited the chitin-induced ROS burst (Fig. 4D), suggesting that PcaRXLR4 and PcaRXLR56 may suppress PTI pathways triggered by different PAMPs. We also evaluated whether PcaRXLR4 and PcaRXLR56 could facilitate *P. cactorum* infection. GFP or PcaRXLRs were expressed in leaves 24 h before *P. cactorum* inoculation. It was found that PcaRXLR4 and PcaRXLR56 significantly enhanced *P. cactorum* infection, resulting in larger lesions in leaves expressing these two RXLRs compared with leaves expressing controls (Fig. 4E).

### Silencing *PcaRXLR4* and *PcaRXLR56* impairs *P. cactorum* infection in *N. benthamiana*

**Fig. 5:**
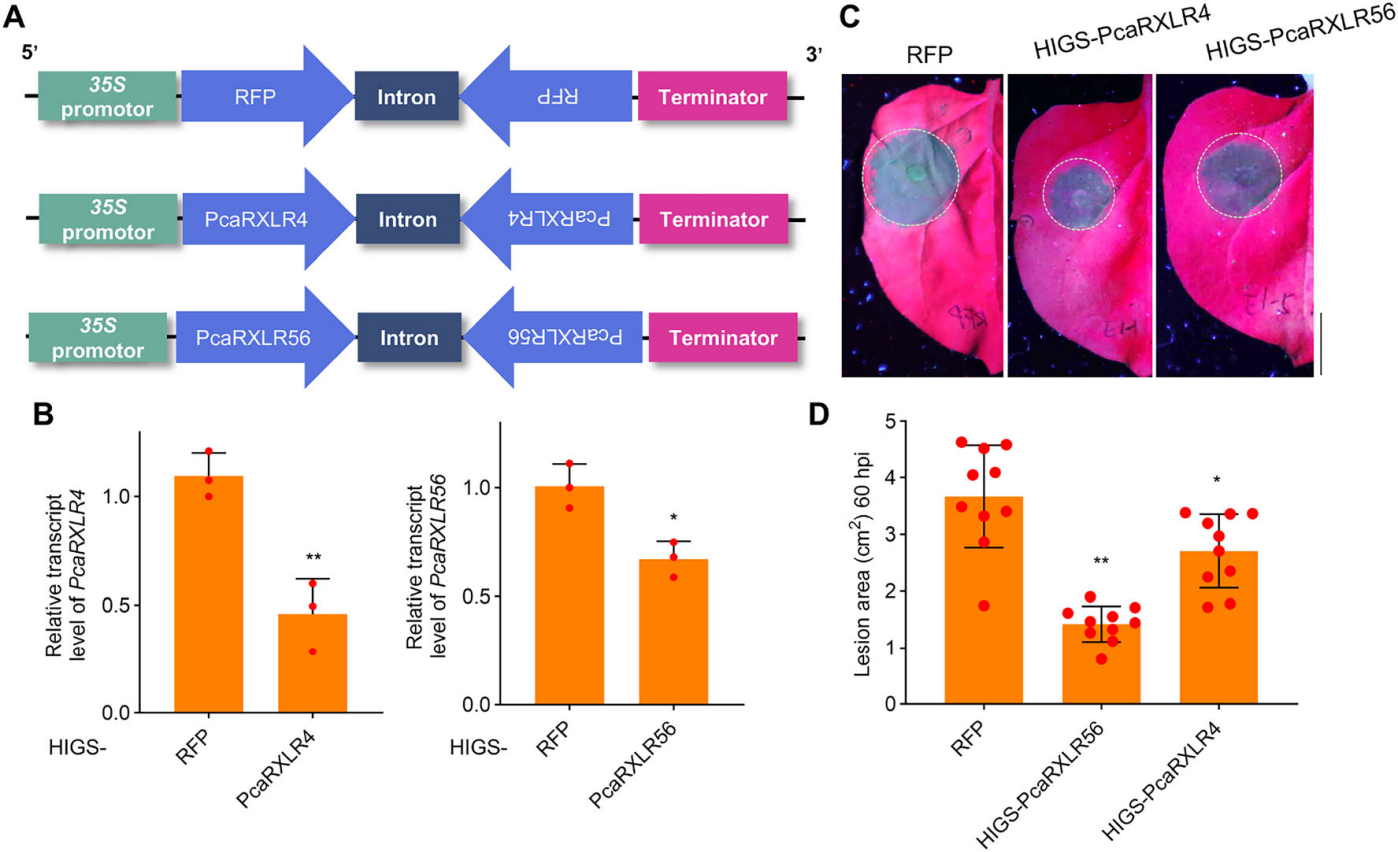
Silencing *PcaRXLR4* and *PcaRXLR56* impairs *P. cactorum* infection in *N. benthamiana*. **A** Schematic map of the three different structures of the designed HIGS vectors. **B** Silencing efficiency of *PcaRXLR4* and *PcaRXLR56 P. cactorum* inoculating *N. benthamiana* plants expressing HIGS vectors. RNA was extracted from mycelium collected from three independent biological replicates (leaves) and pooled for analyzing the silencing efficiency of *PcaRXLR4* and *PcaRXLR56*. Values Data represent mean ± SD of three technical replicates (n = 3; **, *P* < 0.01 compared with the RFP; Student’s *t*-test). **C**–**D** Silencing *PcaRXLR4* and *PcaRXLR56* impairs *P. cactorum* infection in *N. benthamiana* leaves. Indicated constructs were expressed in the leaves of *N. benthamiana*, followed by inoculation with *P. cactorum*. Healthy tissue appears red under UV light while infected tissue appears blue (**C**). Lesion areas were determined by ImageJ (**D**). Data represent mean ± SD (n = 12); significant differences are indicated by lowercase letters, with significance determined by one-way ANOVA at *P* < 0.05). Bar = 1 cm

We employed Host-Induced Gene Silencing (HIGS) technology to knock down *PcaRXLR4* and *PcaRXLR56* in *P. cactorum*. HIGS is a widely adopted method for gene silencing in oomycete species lacking established genetic modification protocols (Hou et al. 2019; Wang et al. 2022b). Specific fragments from *PcaRXLR4* and *PcaRXLR56* were selected and cloned into pCambia2300-intron to generate inverted-repeat constructs (Fig. 5A). All fragments were verified by BLAST analysis to ensure the absence of sequences with 18 or more consecutive identical nucleotides in other *P. cactorum* genes.

The constructs were expressed in *N. benthamiana* leaves, which were subsequently inoculated with *P. cactorum* mycelium. Mycelial samples were collected 24 h post-inoculation. Quantitative PCR analysis revealed that, compared to controls, expression of *PcaRXLR4* and *PcaRXLR56* in *P. cactorum* was reduced by approximately 40–60% in *N. benthamiana* leaves expressing the HIGS constructs, confirming successful gene silencing (Fig. 5B). In pathogenicity assays, the lesion areas caused by *P. cactorum* at 60 h post-inoculation were significantly smaller in leaves expressing HIGS-*PcaRXLR4* or -*PcaRXLR56* compared to control leaves (Fig. 5C, D). These results demonstrate that both *PcaRXLR4* and *PcaRXLR56* contribute to *P. cactorum* pathogenicity. These results indicated that PcaRXLR4 and PcaRXLR56 are virulent effectors that could inhibit PTI.

### PcaRXLR65 and PcaRXLR107 induce HSP90/SGT1-mediated cell death in *N. benthamiana*

**Fig. 6:**
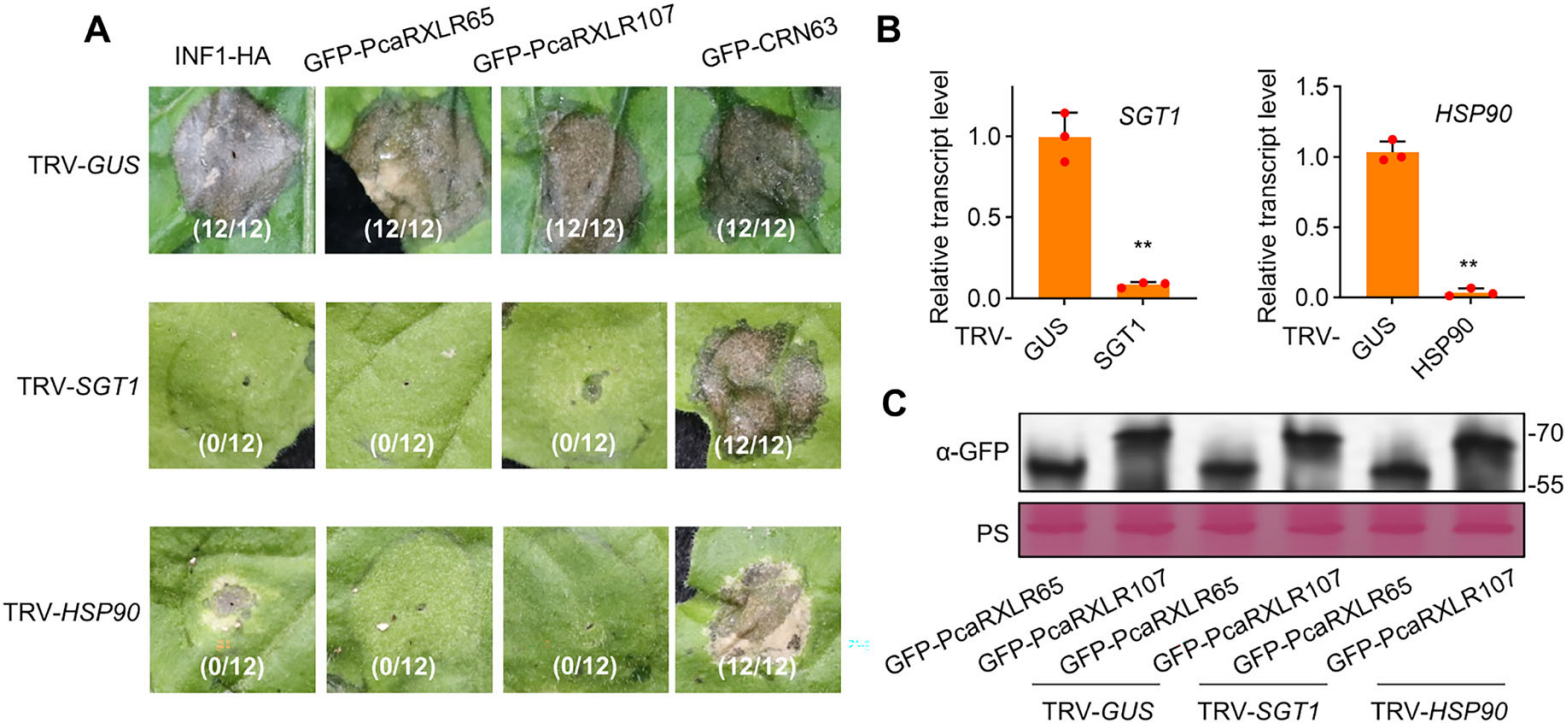
PcaRXLR65 and PcaRXLR107 induce HSP90/SGT1-mediated cell death. **A** Cell death phenotype of PcaRXLR65 and PcaRXLR107 indicated leaves. Indicated expression of proteins on leaves. Images from day 6 post-infiltration (dpi). The numbers indicate the ratio of leaves showing cell death to total leaves analyzed. **B** Silencing efficiency of *SGT1* and *HSP90* in the indicated gene knock-down *N. benthamiana* plants. RNA was extracted from leaves collected from three independent biological replicates (silenced plants) and pooled for analyzing the silencing efficiency of *SGT1* and *HSP90*. Values Data represent mean ± SD of three technical replicates (n = 3; **, *P* < 0.01 compared with the GUS; Student’s *t*-test). **C** Immunoblot analysis. The α-GFP (green fluorescent protein) antibody was used to detect expression of the indicated constructs. Equal loading of each sample is indicated by Ponceau S staining (PS) of the RuBisCO protein

Effectors can induce cell death in host plants, potentially functioning as avirulence proteins recognized by host NLRs, as defense inducers or as toxin-like proteins. To investigate whether conserved atypical PcaRXLRs could trigger cell death, we screened 17 atypical PcaRXLRs and identified that both PcaRXLR65 and PcaRXLR107 induced significant cell death in *N. benthamiana* leaves (Fig. 6A).

To elucidate the underlying signaling pathways, we investigated whether this cell death response required the host proteins SGT1 and HSP90. We silenced *SGT1* and *HSP90* in *N. benthamiana* using Virus-Induced Gene Silencing (VIGS) and confirmed successful knockdown by qPCR (Fig. 6B). PcaRXLR65 and PcaRXLR107 were then expressed in *SGT1*- or *HSP90*-silenced plants. As controls, we used INF1, which induces SGT1- and HSP90-dependent cell death, and CRN63, a *P. sojae* effector that triggers cell death independently of SGT1 and HSP90 (Li et al. 2016). The cell death induced by PcaRXLR65 and PcaRXLR107 was abolished in plants silenced for either *SGT1* or *HSP90* (Fig. 6A), indicating that both proteins are required for the cell death response. Importantly, we found no reduction in PcaRXLR65 and PcaRXLR107 protein levels in *HSP90*- and *SGT1*-silenced plants (Fig. 6C), demonstrating that the compromised cell death response was not due to decreased effector protein accumulation.

## Discussion

RXLR effector proteins are produced and released from oomycetes and are then transferred into host cells to subvert the host immunity. Typical RXLRs contain an RXLR-dEER motif at the N terminus, while atypical proteins display diversity in the dEER region. Here, both typical and atypical RXLRs were identified in *P. cactorum* and were compared and contrasted with proteins from other oomycete pathogens. Fewer RXLRs were found in *P. cactorum* relative to other *Phytophthora* species, possibly due to fewer duplications occurring in the RXLRs of *P. cactorum*. However, the percentage of atypical RXLRs in *P. cactorum* exceeded that in other species, highlighting their significant role in *P. cactorum* pathogenicity. Transcript analysis of atypical RXLR-encoding genes showed that most were expressed, suggesting that they are functional. Moreover, assays in *N. benthamiana* revealed that two atypical RXLRs suppressed the plant defenses, thus enabling *P. cactorum* infection. Additionally, two RXLRs induced necrosis in a manner dependent on the host proteins SGT1 and HSP90. Together, these results demonstrate the critical function of atypical RXLRs in *P. cactorum* and provide insights into their evolution and host interactions.

In contrast to other *Phytophthora* species, relatively few RXLRs were found in the *P. cactorum* genome. Earlier studies have suggested that duplications of effectors facilitate pathogen adaptation to new hosts (Raffaele et al. 2010). For instance, duplication is reported to have shaped the evolution of *P. guiyangense* RXLRs (Ai et al. 2020). *RXLR* genes also cluster closely in *P. ramorum,* implying that clustering may be an oomycete strategy for host adaptation (Win et al. 2007). Interestingly, compared with the substantial clustering of RXLRs in *P. ramorum* and *P. sojae*, minimal grouping occurred in *P. cactorum*. This indicates that fewer duplications in *P. cactorum* RXLRs may account for the smaller RXLR repertoire, suggesting that duplication was not the primary driver of *P. cactorum* RXLR evolution.

Transcriptome analysis of *P. cactorum RXLRs* demonstrated that nearly half of the predicted *RXLRs* were transcribed, also confirming the reliability of the prediction and annotation. Furthermore, atypical *RXLRs* were also transcribed, indicating that they might be functional effectors. Interestingly, atypical *RXLRs* were found to be conserved between different *P. cactorum* isolates. Seventeen atypical *RXLRs* were observed to be expressed in three *P. cactorum* isolates. RXLR is a protein family that evolved quickly. In *P. sojae* isolates, there are only 42 RXLRs among totally 471 members and are considered as core RXLRs that important for infection (Zhang et al. 2019). Some of the conserved atypical PcaRXLRs are also vital for *P. cactorum* colonization.

*P. cactorum* is a destructive pathogen, infecting many crops. However, the mechanism by which *P. cactorum* manipulates plant immunity to facilitate its successful colonization is largely unknown. Here, we identified two RXLR effectors from *P. cactorum* and demonstrated that they were able to suppress plant defense systems. This finding demonstrates the participation of *P. cactorum* cytoplasmic effectors in suppressing host defense reactions. The mechanisms by which these two RXLRs suppress plant immunity require further investigation.

To date, all avirulence genes identified in *Phytophthora* pathogens are RXLR effectors. They can be detected by intracellular NLR receptors, inducing a severe defense response, such as cell death. Here, we found that PcaRXLR65 and PcaRXLR107 could induce the death of *N. benthamiana* leaf cells. Importantly, induction of cell death by PcaRXLR65 and PcaRXLR107 depended on both SGT1 and HSP90, suggesting that unknown NLR(s) may be involved in the recognition of these two RXLRs or the two effectors function as toxin-like proteins or as defense response inducers independent of NLR recognition. Analyzing the behind mechanism could be an interesting work in the future.

In summary, we identified RXLR effectors in *P. cactorum* and focused on the atypical RXLRs. Their evolutionary features, sequence characteristics and expression patterns were analysed in detail. The effector functions of selected *P. cactorum* RXLRs, such as induction of cell death and prevention of host defence, were confirmed experimentally. These findings enhance the understanding of RXLRs in *P. cactorum* and provide a foundation for further investigation into the functions of atypical RXLRs in the interaction between *P. cactorum* and different host plants.

## Materials and methods

### Datasets

The sequenced genomes of *P. ramorum* and *P. sojae* (Tyler et al. 2006) were downloaded from the website of the Joint Genome Institute (http://genome.jgi-psf.org). The sequenced genomes of *Hyaloperonospora arabidopsidis* (Baxter et al. 2010), *P. aphanidermatum* (Adhikari et al. 2013), *P. arrhenomanes* (Adhikari et al. 2013), *P. irregulare* (Adhikari et al. 2013), *P. iwayamai* (Adhikari et al. 2013), *P. ultimum* (Levesque et al. 2010), *P insidiosum* (Rujirawat et al. 2018), *P. guiyangense* (Shen et al. 2019), *S. parasitica* (Jiang et al. 2013), and *P. cactorum* (Chen et al. 2023) were acquired from the National Center for Biotechnology Information (NCBI, https://www.ncbi.nlm.nih.gov/).

### RXLR effector identification

The open reading frames (ORFs) of all genes encoding proteins with a minimum of 50 residues were identified. SignalP (v3) was used for the prediction of signal peptides (SPs), using the criteria of a cleavage site between amino acids 10 and 40 and an HMM prob ≥ 0.9 (Bendtsen et al. 2004). TMHMM (v2.0) was used to predict transmembrane (TM) domains (Krogh et al. 2001). Sequences that contained SPs but lacked a TM were then assessed for the RXLR using a modified regex model described as SP. R.LR. ([ED].[ED][KR]**|**[ED][ED].KR]). All matched protein sequences were identified as RXLR effectors. In the case of redundancy, where overlapping ORFs occurred within a reading frame, only the ORF coding for the longest RXLR protein was used.

### Plasmid construction

Briefly, INF1 (as a control) and indicated PcaRXLRs (without SP, cloned from *P. cactorum*) were cloned into pBinGFP2. For silencing *SGT1* and *HSP90* in *N. benthamiana*, gene fragments were incorporated individually into the pTRV2 vector (Burch-Smith et al. 2004). The primers are shown in Table S3.

### *N. benthamiana* cultivation

*N. benthamiana* were grown in greenhouse (25 °C, 60% relative humidity, 16-h light/8-h dark photoperiod). VIGS-treated *N. benthamiana* plants used in this study were grown in the greenhouse (22 °C, 58% relative humidity, the same light/dark photoperiod.

### RT-qPCR analysis

Total RNA was extracted from *N. benthamiana* leaves using an RNA-simple Total RNA Kit (Tiangen Biotech Co., Ltd., Beijing, China), following the provided directions, reverse-transcribed to cDNA using a HiScript II Q RT SuperMix for qPCR (Vazyme Biotech Co., Ltd., Nanjing, China), and amplified using Real-Time PCR SYBR Premix Ex Taq Kit (Takara Bio Inc., Shiga, Japan) on an ABI Prism 7500 Fast Real-Time PCR system. The reference gene was *N. benthamiana NbEF1α*. Three independent biological replicates were used.

For testing the silencing efficiency of *P. cactorum* in HIGS assay. The constructs for HIGS were expressed in *N. benthamiana* leaves, which were subsequently inoculated with *P. cactorum* mycelium. Mycelial samples were collected 24 h post-inoculation using a hole punch with a diameter of 5 cm. Total RNA was extracted using an RNA-simple Total RNA Kit (Tiangen Biotech Co., Ltd., Beijing, China), following the provided directions, reverse-transcribed to cDNA using a HiScript II Q RT SuperMix for qPCR (Vazyme Biotech Co., Ltd., Nanjing, China), and amplified using Real-Time PCR SYBR Premix Ex Taq Kit (Takara Bio Inc., Shiga, Japan) on an ABI Prism 7500 Fast Real-Time PCR system. The reference gene was *P. cactorum.*

### Transient expression and VIGS

*Agrobacterium tumefaciens* strain GV3101 was used for transformation of the recombinant constructs. After growing for 48 h at 28 °C with shaking at 220 rpm, the culture was centrifuged (4000 rpm, 4 min) and the precipitated cells were resuspended in infiltration medium [10 mM MgCl_2_, 10 mM MES (PH5.7) and 200 μM acetosyringone] to an OD600 value between 0.4 and 0.6. Infiltration of the bacteria was performed using five-week-old leaves from *N. benthamiana*.

For VIGS, the pTRV-RNA1 and pTRV-RNA2 vectors, namely pTRV-SGT1, pTRV-HSP90, and pTRV-GUS (negative control) were transfected into the *A. tumefaciens* strain GV3101 using electroporation. Equal volumes of bacterial suspensions containing pTRV-RNA1 and pTRV-RNA2 were combined and used for inoculation of true leaves of 20-day-old *N. benthamiana* plants cultivated in soil. The plants were then grown for 25 days as described above.

### HIGS

Adjacent 105 nt to 360 nt fragment of *PcaRXLR4* and 354 nt to 699 nt fragment of *PcaRXLR4* were selected from each gene and inserted into pCambia2300-intron to form inverted-repeat constructs. All fragments were checked by BLAST to confirm the absence of stretches of 18 or more nucleotides of identical sequence in the other *P. cactorum* genes. All fragments were amplified and cloned into the vectors and were verified by DNA sequencing performed in Tsingke (Tianjing, China). Primers for HIGS vectors construction are listed in Table S3.

### Oxidative burst assay

Protein-expressing *N. benthamiana* leaves were cut into circles (5 mm diameter), placed in 200 µL of water in 96-well plates, and incubated overnight. The following day, 200 µL of luminescence detection buffer, containing 100 mM luminol and 20 mg/mL horseradish peroxidase, together with 1 mM flg22 or 1 mM chitin, was added and the luminescence was recorded with a microplate reader (BioTek) for 30 min.

### Phylogeny tree

For constructing the phylogeny tree, sequences were aligned using the MUSCLE software (Edgar 2004). Phylogenetic trees were constructed using the MEGAX software (https://www.megasoftware.net/) with Neighbor-Joining method (Bootstrap = 1000, p-distance, pairwise deletion).

## Supplementary Information

Below is the link to the electronic supplementary material.

Supplementary file1 (XLSX 72 KB)

Supplementary file2 (XLSX 376 KB)

Supplementary file3 (XLSX 10 KB)

## Data Availability

All data supporting the findings of this study are available within the article and supplementary files.
